# Management of Retained Genital Piercings: A Case Report and Review

**DOI:** 10.1155/2017/2402145

**Published:** 2017-02-19

**Authors:** Laura J. Moulton, Amelia M. Jernigan

**Affiliations:** ^1^Cleveland Clinic Foundation, Ob/Gyn and Women's Health Institute, 9500 Euclid Ave. A81, Cleveland, OH 44195, USA; ^2^Division of Gynecologic Oncology, Ob/Gyn and Women's Health Institute, 9500 Euclid Ave. A81, Cleveland, OH 44195, USA

## Abstract

The prevalence of genital piercing among women is increasing. As the popularity increases, the number of complications from infection, injury, and retained jewelry is likely to rise. Techniques to remove embedded jewelry are not well described in the literature. The purpose of this report was to describe a case of a patient with a retained clitoral glans piercing, discuss a simple technique for outpatient removal, and review current evidence regarding associated risks of clitoral piercings. A 24-year-old female presented to the emergency department with an embedded clitoral glans piercing. Local anesthetic was injected into the periclitoral skin and a small superficial vertical incision was made to remove the ball of the retained barbell safely. In conclusion, among patients with retained genital piercing, outpatient removal of embedded jewelry is feasible. While the practice of female genital piercing is not regulated, piercing of the glans of the clitoris is associated with increased injury to the nerves and blood supply of the clitoris structures leading to future fibrosis and diminished function compared to piercing of the clitoral hood.

## 1. Introduction

Genital piercing is increasing in frequency among many demographic groups. Genital piercings are unique as they may have a functional role in addition to aesthetical value, with some women reporting heightened sexual arousal and increased orgasm potential after clitoral piercings [1]. In women, clitoral piercing may play a therapeutic role in overcoming past genital trauma or dysfunctional sexual relationships [2]. As the popularity for genital piercing grows, the number of related complications is likely to increase. In a survey of men and women with genital piercings, 53% of participants reported complications as a result their procedure [3]. Bleeding, infection, allergic reactions to jewelry, keloid formation, and scarring have been reported among the complications of female genital piercings [1, 4, 5]. Embedded jewelry after clitoral piercing is rare and the management is not well described in the literature. In this case report, we describe a case of a woman who presented to the emergency department with an embedded clitoral piercing, which was removed with a simple outpatient procedure.

## 2. Case

**Figure 1:**
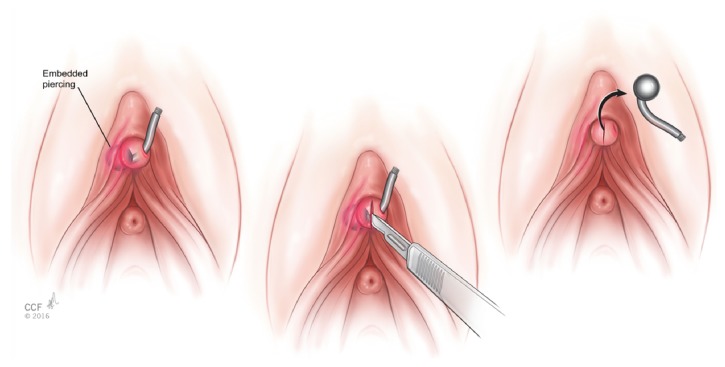
Illustration of removal of embedded clitoral glans piercing removed successfully with an outpatient procedure.

A 24-year-old female presented to the emergency room with pain at the site of her clitoral piercing for 1 day. She reported that earlier that day, she noted that the piercing did not move freely on with touching the area. The patient was sexually active one day prior. The patient's own attempts to remove the piercing were unsuccessful due to pain. She had also returned to the tattoo parlor where the piercing was performed 8 months earlier who recommended she seek medical care. On examination, the labia were normal without lesions. There was a straight metal barbell protruding at a perpendicular angle from the glans of the clitoris. On closer inspection, one of the removable balls was missing from the barbell externally and on palpation, the other ball attached to the straight barbell was appreciated underneath the glans of the clitoris. There was no drainage, bleeding, or evidence of infection. There was no evidence of scarring or keloid formation at the site of the piercing. The patient was unable to tolerate initial attempts to remove due to pain. She gave consent for attempted removal of the piercing in the emergency department. She was premedicated with 0.2 milligrams of intramuscular hydromorphone and the area was thoroughly cleansed with betadine. Then, 3 cc of 1% lidocaine without epinephrine was injected into the periclitoral skin and tested to ensure analgesia. A #11 scalpel was used to make a 2-millimeter superficial incision within the nonkeratinizing squamous epithelium overlying the clitoral glans over the embedded ball of the piercing (Figure 1). The embedded end of the piercing was then removed easily with gentle traction with forceps on the free end. The area was hemostatic after the procedure and did not require any sutures. She was discharged home from the emergency department with follow-up in gynecology clinic.

## 3. Discussion

Genital piercing is not a new phenomenon. There are historical records of male genital piercing from as early as the 1st century B.C., and this practice has been alluded to in writings from the Mayan, Victorian, and World War II eras [6]. While genital piercing is not a new practice, information about the safety and complications did not reach the healthcare literature until the mid-1990s [3]. Recent reports estimate that female clitoral piercing is increasing among many demographic groups [1, 3–6]. Despite its longevity throughout time, there is still much to be learned about genital piercings, especially in women.

The practice of female genital piercing is not standardized. Common anatomical locations for female genital piercings include the labia majora, labia minora, and clitoris. The clitoris may be pierced through the prepuce, either horizontally or vertically or through the glans of the clitoris. Alternative female genital piercing locations that have been described include a vertical clitoral piercing exiting superiorly on the mons pubis, placement of a ring entering through the urethra and exiting between the urethral and vaginal openings (“Albertina Piercing”) [7, 8], and posterior fourchette piercing. There are several complications of genital piercings that have been well documented in the literature, including bleeding, infection, trauma, and development of scar tissue. In a review by Anderson et al. they reported that genital piercing complications occur due to 4 reasons: poor technique during insertion, lack of adequate hygiene and care for the piercing, body changes after long-term jewelry wear, and partner damage [7].

For clitoral piercings specifically, piercings through the clitoral hood or prepuce are associated with decreased rates of infection, injury to the clitoris, and fibrosis. Genital piercing of the clitoral glans has been associated with higher rates of loss of sensation and diminished sexual function due to increased risk of damage to the nerves and vascular structures that supply of clitoris (dorsal artery and vein and deep artery and vein) [8].

Traumatic injuries occurring at the time of insertion or afterwards, such as during sexual intercourse, are not uncommon [7, 8]. Surgical repair is generally preferred to ensure that the delicate anatomic structure is retained [4]. However, a recent case report described the usage of 2-octyl cyanoacrylate (Dermabond) after a traumatic clitoral avulsion during a sexual encounter with full return of orgasmic function [9]. Ensuring good hygiene at the site of the piercing is essential. Chronic irritation and inflammation can lead to keloid and granuloma formation overtime. This may be prevented with use of stainless steel jewelry exclusively and meticulous self-care practices [4]. In patients with scar tissue or keloids, surgical removal or intralesional steroids have been helpful [1, 4]. While retained or embedded jewelry is reported as a known complication of genital piercings, there is little published information about patients who are at risk and management strategies. To our knowledge, this is the first report detailing the removal of an embedded clitoral piercing.

Embedded jewelry from piercings has been reported in other areas of the body, most commonly for lingual piercings. Piercings of the tongue and clitoris are alike as barbell jewelry is commonly used in both which consists of a central stud with two peripheral balls [4, 8, 10]. The length of the central bar should exceed the thickness of the tongue or clitoris to permit free movement and prevent the jewelry from becoming trapped underneath the skin [10]. If one of the peripheral studs on the barbell becomes loose and the jewelry length is inadequate, or the tissue is too wide, as in the case of swelling, this may cause the jewelry to become embedded. Several case reports have reported presence of an embedded barbell within the body of the tongue occurring several months after the piercing first occurred [10, 11]. In these cases, surgical excision of the embedded jewelry was performed under both general and local anesthesia. Similar to our case, small incisions were made on the dorsal surface of the tongue and the jewelry was removed intact with no long-term sequelae [10, 11]. A limitation of this report is that the patient was not followed longitudinally to assess for long-term sequelae of the clitoral piercing and removal procedure, including scarring, diminished sexual function, inability to achieve orgasm, dyspareunia, or vaginal pain. Patients should be counseled on the possibility of these outcomes prior to undergoing a removal procedure. In patients with clitoral piercings, the barbell length should be long enough to ensure free movement and patients should ensure that the two peripheral balls that secure the jewelry are screwed tight to avoid the jewelry becoming embedded.

Healthcare providers should be aware of the increasing prevalence of genital piercing and the complications inherent to this practice. Complications after genital piercings are common, reported in over 50% of patients who had genital piercings. While the practice of female genital piercing is not standardized or supported by a wealth of evidence based literature, prior studies do suggest that piercing of the glans of the clitoris is associated with higher rates of injury to the neurovascular structures leading to future fibrosis, decreased function, and injury [7, 8]. Therefore, clitoral piercings through the prepuce should be considered superior given the lower rate of injury and risk of diminished sexual function and patients should be aware of these risks prior to the piercing procedure. Retained jewelry is a known complication of genital piercings, but there is a paucity of published information on how to manage this problem. The best removal method in these delicate and sensitive areas must be tailored to the patient, the embedded object, and the physician's skill set. In this case, removal was performed safely in an outpatient setting avoiding the morbidity of general anesthesia and the expense of operative management. As the popularity for genital piercings increases, clinicians should be prepared to handle the potential complications.
